# Acknowledgment to the Reviewers of *NeuroSci* in 2022

**DOI:** 10.3390/neurosci4010005

**Published:** 2023-01-18

**Authors:** NeuroSci Editorial Office

**Affiliations:** MDPI AG, St. Alban-Anlage 66, 4052 Basel, Switzerland

High-quality academic publishing is built on rigorous peer review. *NeuroSci* was able to uphold its high standards for published papers due to the outstanding efforts of our reviewers. Thanks to the efforts of our reviewers in 2022, the median time to first decision was 15 days and the median time to publication was 41 days. Regardless of whether the articles they examined were ultimately published, the editors would like to express their appreciation and thank the following reviewers for the time and dedication that they have shown *NeuroSci:*
Albert Van Der ZwanMarika SyrrouAna Quelle-RegaldieMark B. FramptonAndré Pimenta FreireMichela BossaAndrey Y. VinokurovMiren AltunaAngelo TorrenteMuthukumar KannanAnnachiara CavaliereNadia D’AmbrosiAnne ParkinsonNeil M. FournierAntonio PalladinoNicolas RouleauAyush RanawadeNoemí Cárdenas-RodríguezAzzurra StefanucciOlga E. DickBarbara BarunOmid MirmosayyebBartłomiej SzulczykPablo BarttfeldBertold RennerPatricia K. CoyleBissera PilichevaPeter BoltucCano-Ramírez HugoPeter MachnikCarolina Sitges QuirosPietro CacialliChander Singh DigwalPiotr PoznanskiCharlotte Von GallRobert FriedmanChitra MandyamRonald BonnstetterDawon KangRyan O. KellemsDebadatta DashSagrario Martín-AragónDomenico SolariSally EsmailDominique DurandSanghoon LeeEkaterina YurchenkoSangjune KimEleonora VirgilioSara Mederos CrespoFrancesco AsciSaurabh PandeyGábor JancsóSebastián HormigoGaetano D. GargiuloShalini DograGiovanna RigilloShangru LyuGiovanni FedericoSimona ScainiGisella VetereStanislav M. CherepanovGraham PluckSusan L. CampbellHeather BloomSusanna PietropaoloJacopo FalcoTaisia RolovaJae Yon WonTaiyong LiJanani ParameswaranTakuma InagawaJishnu KrishnanTino Emanuele PoloniJohannes LevinTsutomu SawaiJon MallattTunde CsepanyJorge Arturo Santos LópezUdaiyappan JanakiramanJulien BogousslavskyValentyn OksenychKathryn ChadmanVanesa Soto-LeónKavita SinghVenediktova NatalyaKeld-Erik BygVijai KrishnanKenneth NormanVincent PonsKonstantin KorotkovVirginie RappeneauKristine M. KrajnakWael El-DeredyLászló VécseiWei ChenLaura Jiménez-SánchezWilly CarrasquelLavazza AndreaWladimir Bocca Vieira De Rezende PintoLi ZengXiaodong MaLucia BuccarelloXueying WangManjari GuptaYajun XieMarco RinaudoZurina HassanMarcos Brioschi



